# Challenges and Opportunities in Calibrating Low-Cost Environmental Sensors

**DOI:** 10.3390/s24113650

**Published:** 2024-06-05

**Authors:** Naga Venkata Sudha Rani Nalakurthi, Ismaila Abimbola, Tasneem Ahmed, Iulia Anton, Khurram Riaz, Qusai Ibrahim, Arghadyuti Banerjee, Ananya Tiwari, Salem Gharbia

**Affiliations:** Smart Earth Innovation Hub (Earth-Hub), Atlantic Technological University, F91 YW50 Sligo, Ireland; sudha-rani.nalakurthi@atu.ie (N.V.S.R.N.); s00232668@atu.ie (I.A.); tasneem.ahmed@research.atu.ie (T.A.); iulia.anton@atu.ie (I.A.); khurram.riaz@research.atu.ie (K.R.); qusai.ibrahim@mail.itsligo.ie (Q.I.); arghadyuti.banerjee@research.atu.ie (A.B.); ananya.tiwari@research.atu.ie (A.T.)

**Keywords:** low-cost sensors, water quality, air quality, calibrations

## Abstract

The use of low-cost environmental sensors has gained significant attention due to their affordability and potential to intensify environmental monitoring networks. These sensors enable real-time monitoring of various environmental parameters, which can help identify pollution hotspots and inform targeted mitigation strategies. Low-cost sensors also facilitate citizen science projects, providing more localized and granular data, and making environmental monitoring more accessible to communities. However, the accuracy and reliability of data generated by these sensors can be a concern, particularly without proper calibration. Calibration is challenging for low-cost sensors due to the variability in sensing materials, transducer designs, and environmental conditions. Therefore, standardized calibration protocols are necessary to ensure the accuracy and reliability of low-cost sensor data. This review article addresses four critical questions related to the calibration and accuracy of low-cost sensors. Firstly, it discusses why low-cost sensors are increasingly being used as an alternative to high-cost sensors. In addition, it discusses self-calibration techniques and how they outperform traditional techniques. Secondly, the review highlights the importance of selectivity and sensitivity of low-cost sensors in generating accurate data. Thirdly, it examines the impact of calibration functions on improved accuracies. Lastly, the review discusses various approaches that can be adopted to improve the accuracy of low-cost sensors, such as incorporating advanced data analysis techniques and enhancing the sensing material and transducer design. The use of reference-grade sensors for calibration and validation can also help improve the accuracy and reliability of low-cost sensor data. In conclusion, low-cost environmental sensors have the potential to revolutionize environmental monitoring, particularly in areas where traditional monitoring methods are not feasible. However, the accuracy and reliability of data generated by these sensors are critical for their successful implementation. Therefore, standardized calibration protocols and innovative approaches to enhance the sensing material and transducer design are necessary to ensure the accuracy and reliability of low-cost sensor data.

## 1. Introduction

Climate change is a significant challenge for environmental sustainability [1,2], and to address this issue effectively, it is crucial to advance scientific knowledge by collecting and comprehending information on various aspects related to climate change [3]. This requires a comprehensive understanding of the global environmental system and data collection is the most crucial part of this process.

The environment is facing significant challenges worldwide, particularly in terms of pollution which substantially degrades it. These challenges are largely due to a combination of factors, including population growth, the ageing of infrastructure, the impacts of climate change, and ongoing global development [4,5]. Environmental pollution including water, air and soil pollution has become prevalent globally. More research needs to be conducted to effectively monitor and understand the sources, concentrations, and effects of environmental pollutants to aid policymakers and citizens in developing strategies for preventing pollution and protecting the environment, particularly in vulnerable regions.

Thus, there is an urgent need to develop a more effective and rapid method of monitoring environmental pollution given that the traditional methods of collecting data and samples which often involve laboratory analysis are expensive, time-consuming, and labour-intensive. Also, traditional data collection methods are not real-time and lack the fast data collection and dissemination which are needed for an effective and timely response to protect the environment [6,7]. The traditional method of monitoring the environment, e.g., water and air quality parameters, involves manual collection of samples from different areas and carrying out several laboratory analyses to process, analyse and characterise samples. These traditional methods for environmental monitoring are now seen as inadequate for effectively monitoring the environment, partly because they are also prone to human error [6,8]. This has led to several researchers emphasising the need for developing low-cost, robust, and standard methods and sensors for identifying and quantifying pollutants in the environment.

Environmental attributes such as the quality of water, air, and soil are usually observed through traditional sensors located at established monitoring stations. These conventional in-situ methods, employing stationary sensors, come with limitations in data resolution and necessitate intensive training and maintenance. Meanwhile, when using satellite data, challenges arise due to disparities in spatial and temporal scales compared to environmental occurrences [9]. Furthermore, the need for increased data collection density has surged over the last two decades, driven by population growth and escalating levels of air and water pollution.

Recent advancements in digital electronics, wireless communication technologies, and sensor manufacturing [10] have generated a growing demand within the field of environmental science for low-cost sensor networks (LCSNs). These networks are increasingly valuable for addressing both fundamental research inquiries and practical management challenges [11,12]. This shift in approach is driven by the accessibility of low-cost sensors (LCSs) equipped with user-friendly technologies and calibration methods that yield data with enhanced spatial resolution [13,14,15]. Several factors, including the decreased costs of microcontrollers for sensors, environmental sensor components, and straightforward communication modules, have played pivotal roles in bringing about this shift. Additionally, the expanded spatial coverage afforded by LCSs enables the generation of fresh insights into environmental dynamics [16].

The term ‘low-cost’ sensor does not define a specific price range, as the cost can vary depending on the specific parameters being measured. It can be defined as a sensor that is relatively inexpensive to produce, purchase, and maintain compared to other sensors with similar functionalities [14].

Low-cost sensors are the latest and most innovative technology used in monitoring water and air quality in real time. The use of low-cost sensors for environmental sensing and monitoring is increasing due to the availability and affordability of low-cost sensors, internet facilities, and cloud computing services [8,17]. Low-cost sensors also require fewer human interventions to operate and thus are less biased compared to traditional techniques and can be deployed in remote and inaccessible locations.

Wireless network sensors, for example, have become popularly employed by researchers for environmental monitoring of factors such as water quality parameters e.g., temperature, pH, dissolved oxygen, turbidity, water flow rate, and conductivity [18,19]. They have also been used to measure air quality parameters such as particulate matter, carbon monoxide, and nitrogen dioxide [20,21]. For example, in the past 10 years, studies such as [6,22] have attached water and air quality sensors, respectively, to Arduino controllers; an open-source, user-friendly, and simple platform to measure and monitor water quality parameters including dissolved oxygen, pH, temperature, nitrates, and turbidity in their studies.

The real-time water quality data from the above studies were acquired, processed, and automatically transmitted through Internet of Things (IoT) systems. A network of low-cost sensors that can collect real-time data will aid in the detection and understanding of the sources and pathways of pollution in the environment large and remote. This will be significant in effectively modelling and monitoring the vulnerability of human health and the ecosystem to environmental pollution.

However, any sensor that is more affordable than the instrumentation needed to meet regulatory requirements for the parameter under study is categorized as low-cost [22]. In this context, the cost of sensors typically increases when additional components such as microprocessors, data loggers, memory cards, batteries, and display units are incorporated.

The increased adoption of low-cost sensors (LCSs) in recent studies can be attributed to the user-friendly nature of these sensors, which allows for a cost-effective expansion of spatial coverage that has been traditionally limited. While the existing literature acknowledges the value of LCSs as a valuable addition to the commonly used measurement tools, it consistently highlights the potential for sensor misuse leading to more frequent inaccuracies. It is important to note that data collected from these sensors are indicative of specific locations and their ambient conditions, suggesting underlying factors affecting sensor measurements. In essence, the selectivity of a sensor refers to its ability to differentiate between the intended target and any interfering elements [23]. For example, a gas sensor designed to detect one type of particle often exhibits sensitivity to other particles, which can interfere with the accurate measurement of the target pollutant or particle. This phenomenon is known as sensor cross-sensitivity and can be assessed by exposing the sensor to other pollutants [24].

A standard reference sensor tends to show a higher sensitivity to particles, therefore is more precise, and more selective to measure a specific variable of interest. Therefore, according to WMO reports, low-cost sensors should be used under established quality assurance and quality control protocols [25,26,27]. Further, a more precise calibration approach will be attained with selectivity and sensitivity of a low-cost sensor at any location. Therefore, to use an LCS instrument, a published standard set of criteria must be followed which should be provided by regulatory agencies. Selectivity and sensitivity are two essential factors to take into account while considering an LCS for any given investigation.

Low-cost sensors with higher spatial resolution can provide us with better regional accuracy and offer us even more liberty to choose the variables that are appropriate for the region. The existing literature demonstrates, however, that low-cost sensors suffer from significant uncertainties because of large data outliers, weak correlations, and low data precision [28,29]. The selectivity of the sensors may improve the evaluation, but more thorough calibration procedures that address the difficulties that have been raised can produce improved results.

Sensor calibration is the process of comparing the output of the instrument or sensor under test against the output of an instrument of known accuracy when the same input is applied to both instruments [30] by developing a mathematical function that describes the relationship between the uncalibrated variables and the reference [28]. However, the relationship between uncalibrated and reference is not a direct proportion; there exists the influence of multiple other parameters. Therefore, the calibration function can be improved by utilising cross-sensitive parameters that influence the parameter of interest. Automatic and semi-automatic calibration methods are two calibration methods that are largely used for LCSs [13,28,31,32]. This review article aims to answer several questions related to the increasing popularity of low-cost sensors as an alternative to high-cost sensors. The article explores why selectivity and sensitivity of sensors are crucial factors in low-cost sensors, and how calibration functions can improve accuracy. Additionally, the article discusses ways to enhance the accuracy of sensors, providing insights into the development of low-cost sensing technologies.

## 2. Literature Survey Approach

**Figure 1:**
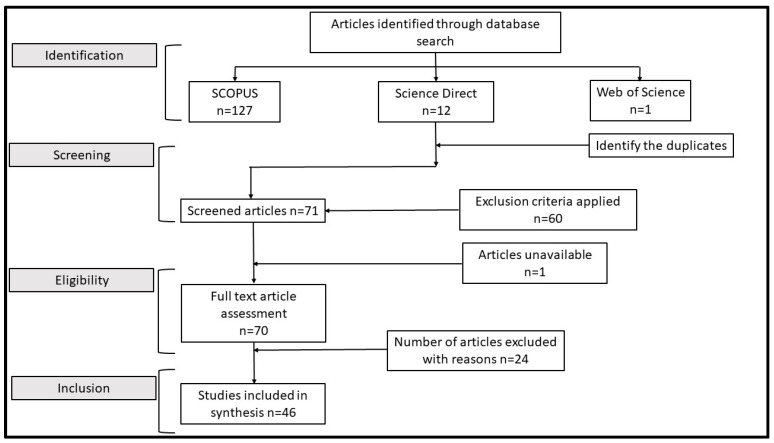
Flowchart for the selection and screening of the articles.

The literature survey is conducted in a structured approach which covers identification, screening, eligibility assessment, and final inclusion. The PRISMA Statement, which outlines the Preferred Reporting Items for Systematic reviews and Meta-Analyses, was the principal criterion used in this study. PRISMA can be defined as guidelines that provide a structured framework for authors to follow when writing and reporting systematic reviews and meta-analyses [33]. Its use by Cochrane Collaboration defines the systematic review as “an examination of a clearly formulated questions that uses systematics and explicit methods to identify, select, and critically appraise relevant research and to collect and analyse data from the studies that are included in the review. Statistical methods may or may not be used to analyse and summarise the results of the included studies” [34,35]. The primary search criteria applied for identification of the articles in each database are as follows: “(“water quality” or “air quality” AND ‘low-cost sensors’ AND “calibration”)” and with time-period 2013 to 2022. The number of articles identified from the databases SCOPUS, Science Direct, and Web of Science are 127, 12, and 1, respectively. The returned articles were uploaded to the Rayyan online platform [36] for screening and preselection of publications for further review of articles. We excluded papers that were not in English, unpublished, or duplicates, resulting in 70 papers being selected for further review. In the subsequent phase, eight of the authors independently evaluated the title, abstract, and conclusions of these 70 papers based on specific inclusion and exclusion criteria detailed in Table 1 to determine their relevance. This process identified 46 relevant papers and a thorough full-text review of the 46 shortlisted papers was conducted. To write this study, we looked at more than just the aforementioned papers and book chapters to determine the types of sensors for each relevant parameter since there are numerous sensors made explicitly for monitoring specific parameters. The PRISMA diagram as applicable to this systematic review study is shown in Figure 1.

## 3. LCSs for Monitoring Air and Water Quality

This article looks at the calibration and validation methods of low-cost sensors with exclusive focus on water and air quality sensors. Table 2 provides a number of selected key studies related to the use of LCSs on air and water quality. The literature shows that in the past decade, the use of LCSs for air-quality assessment has improved rapidly.

### 3.1. LCSs for Air Quality

The selection of LCSs for air quality depends upon the parameter of interest and its characteristic. For instance, sensors for particulate matter (PM) and gaseous pollutants (GL) are different. Furthermore, the cross-sensitivity of the parameter also plays a major role in selecting the sensor. International and national level health organisations have created several protocols for standardised pollutants in the air. For example, the World Health Organisation (WHO) has updated a global air quality guideline for both particulate matter and gaseous pollutants in the air; in the European Union (EU), as part of the ‘European Green Deal’ proposed directives that align with WHO standards, the directive 2011/850/EU [65] is the most recent legislation passed to reduce pollution concentration thresholds. Sensors for measuring air quality can be broadly divided into two groups: (1) sensors for estimating particulate matter concentrations (PMx), and (2) sensors for estimating gaseous contaminants in ambient air. In the sections that follow, we go over them.

#### 3.1.1. LCSs for Particulate Matter

PM is a mixture of airborne solid particles and liquid droplets that can be inhaled with air. Particle mass concentration (Pmass) and particle number concentration (Pnum) are the two basic metrics used to measure atmospheric particulate matter (PM). Pnum is the number of particles in a given volume (particles/cm^3^), and Pmass is the mass of the particles in a given volume (typically g/cm^3^) [66]. PM in general is characterised by its shape, size, and composition (Table 3). The diameter of the particle sub-categorises PM; for example, PM2.5 and PM10 are for particulate matter of diameter 2.5 and 10 micrometres, respectively. The particle concentration (either mass or number) is measured throughout a range of different particle sizes and is referred to as the particle size distribution (Psd).

Currently, a typical commercially available LCS for PM sensing uses the light-scattering principle, with the sensor consisting of three major components: a light emitting diode, photo-transistor, and a lens to focus the diode light [67,68].

The common reference/validation techniques for LCSs monitoring PM are ‘Tapered Element Oscillation Microbalance’ (TEOM) and ‘Beta Attenuation Monitor’ (BAM), both of which measure properties directly associated with Pmass [69].

#### 3.1.2. LCSs for Gaseous Pollutants

Gaseous pollutants include the following pollutants in their gaseous state emitted by, for example, an engine: carbon monoxide (CO), total hydrocarbons (HC), oxides of nitrogen (NOx), and other greenhouse gases (GHG); NOx being nitric oxide (NO) and nitrogen dioxide (NO_2_), expressed as NO_2_ equivalent, and GHG includes carbon dioxide (CO_2_), methane (CH_4_), and nitrous oxide (N_2_O) [70,71,72]. The gas sensors detect the presence of these pollutants (gas concentrations) in the environment using different sensing materials. The main objective of gas sensor development is to establish an array of multifunctional gas sensor technologies that can monitor air pollution at a low cost and be used to create an electronic nose [73]. Different types of gas sensors include electrochemical sensors, metal-oxide semiconductors, catalytic combustion type, acoustic-wave based, and optical gas sensors [73,74] and the selection depends on the gas types, which can be flammable, combustible, and toxic. These sensors are chosen for their affordability, portability, elegant design, limited sensitivity, and selectivity, and the requirement for extra equipment [75] during use. Among them, metal-oxide semiconductor sensors are particularly popular due to their several unique features, such as high sensitivity, rapid response and recovery times, simple manufacturing process, robust stability, easy operation, and low expense [74,75]. Table 4 lists several sensors used for air-quality measurements. PurpleAir is the most used LCS for air-quality parameters due to its easily accessible and cost-effective approach.

### 3.2. LCSs for Water Quality

The quality of water determined by its chemical, physical, and biological properties plays an important role in human health. Monitoring water characteristics including conductivity, pH, salinity, temperature, dissolved oxygen, residual chlorine, and turbidity is essential to maintaining its quality; therefore, water quality monitors are widely used.

Just like in air-quality monitoring, international and national-level health organisations have created several protocols for a standardised range for monitoring water quality. For instance, the Guidelines for Drinking Water Quality (GDWQ) are produced by the World Health Organization (WHO) through regular revisions, of which the most recent is the GDWQ 4th edition. Europe established its own water quality norms by adapting from WHO guidelines known as EU Water Framework Directives (WFD), the most recent being Directive 2006/118/EC [65]. The challenge of maintaining water quality standards is great, and continuous monitoring using a conventional approach is cost-effective and often unable to produce real-time data. Low-cost water quality sensors can overcome this constraint whilst enhancing the spatial density of data. Primary cost components of water quality sensors are designing, installing sensors with power supply utility, communication equipment, access, lighting, security, and environmental conditions of the location.

Two primary approaches for water quality measurement are direct measurement of constituents, and surrogate measurement which are chemical concentrations that indicate the presence of undesired contaminants in the water [78,79]. The following are the most common water quality sensors used to measure key parameters:

#### 3.2.1. Chlorine Residual Sensor

The most popular method of disinfection to lessen water contamination is chlorination. The theory of chlorination is straightforward: When chlorine comes into direct contact with microorganisms in water, it destroys their cellular structure, causing disinfection. Monitoring residual chlorine, which refers to the effective chlorine remaining in water after chlorination, is generally essential to mitigate the risk of chlorine residuals [80]. Although adding a lot of chlorine to the treated water will increase disinfection efficiency, doing so can also cause unpleasant odours, formation of a lot of carcinogenic disinfection by-products, faster distribution system corrosion, and certain health hazards [80,81]. Electrochemical sensors (amperometry and ion-selective electrodes), spectrophotometric sensors (colourimetry and fluorescence), and biosensors are the three main chlorine residual monitoring devices Amperometry sensors, which track changes in current, are the most economical and widely used sensors [82,83]. Moreover, these sensors trigger less with the presence of dissolved oxygen, temperature, pH, and other oxidants than do biosensors and fluorescence sensors.

#### 3.2.2. Total Organic Carbon (TOC) Sensor

TOC measures organic compounds in pure water and aqueous systems and is primarily used in treating wastewater and testing drinking water contamination. The fundamental methods for measuring TOC are based on organic matter oxidation to detect CO_2_ through conductometry and IR spectroscopy [84,85]; however, the process is time-consuming and expensive. Through low-cost sensors it is possible to monitor TOC regularly, rapidly, and with affordability. Campanella et al. 2002 [85] developed a sensor that measures the amount of CO_2_ created by the UV-assisted photodegradation of organic matter which is improved by nanosized TiO_2_ (anatase). TiO_2_ anatase is a colourless, metastable mineral form of titanium dioxide, which is a suitable photocatalyst in the photodegradation of toxic organic molecules due to its high activity, nontoxicity, and chemical inertness [86], and it is widely regarded as the most suitable photocatalyst for TOC contamination studies [86,87,88].

#### 3.2.3. Turbidity Sensor

Turbidity is the most highlighted parameter, which is also known as haziness of a fluid due to suspended solids [89]. The WHO [90] standard for turbidity in ideal drinking water is below 1 NTU (Nephelometric Turbidity Units), as higher levels of turbidity in water produce favourable conditions for contagious pathogens [91]. As in other water quality measures, the fundamental approach to turbidity monitoring is through laboratory analysis due to its reliability and accuracy. However, these products are economically unviable at large scale, therefore for such products spatio-temporal scales reduce drastically, which is not viable for continuous monitoring. Further, these systems require significant preparation and regular management.

Low-cost sensors filling these gaps and mostly used for turbidity are developed with other sensing parameters such as dissolved oxygen, Ph, phosphorous, etc. Low-cost turbidity sensors typically use transmitted light detection (optical sensor) to monitor the haziness of water, though the accuracy and reliability of these sensors can be lower [92].

#### 3.2.4. Conductivity Sensor

Significant increases in water conductivity indicate that the water is contaminated, unsafe for drinking, and may harm aquatic creatures. Conductivity comes under physical water quality parameters like turbidity, hardness, and temperature. With advancing technologies, sensors that measures the conductivity of water can be made rapidly using materials that are readily available; however, the price of a conductivity sensor is still too costly for good coverage of spatial resolution [93].

#### 3.2.5. pH and ORP Sensors

The pH of water indicates alkalinity characteristics, whereas ORP (Oxygen-reduction Potential) gives an insight into the level of oxidation/reduction reactions occurring in the water. The pH for drinking water should be between 6.5 and 8.5, whereas ORP is not a mandatory parameter according to WHO and EU standards. However, the ORP value is a valuable parameter to estimate the physicochemical properties of water [94]. The ORP is primarily useful to check the oxygen reductions happening in water due to contamination. Electrodes are usually used to analyse these two parameters. Both parameters come under the inorganic category [95] and are inversely proportional to each other, which means as pH decreases, ORP increases, and vice-versa [94].

Most water quality parameters are measurable with higher accuracies using conventional laboratory assessments. However, this time-consuming task is not feasible for monitoring of large spatial networks and real-time values. With the advancements in sensor technologies, an overarching approach has been developed to study a selected range of parameters depending upon the geographical location, risks, and usual contamination history. Several attempts have been made to identify a range of water quality parameters with sensor technology; nevertheless, Internet of Things (IoT) has been the most recent advancement in continuous monitoring of water quality. Sensing of parameters like pH, turbidity, temperature, conductivity, and dissolved oxygens are regularly monitored using IoT. [96] developed IoT to track water quality using Thing Speak (IoT technology) which sends data from numerous sensors to Arduino via the cloud.

Lakshmikantha et al. 2021 [97] introduced LED additions to an IoT-based water monitoring system which is connected to a Raspberry Pi using Java. Several sensors were used to determine the range of water quality, and accordingly these LEDs lit up. Salunke & Kate 2017 [98] developed a sensor network with the Intel Galileo Gen 2 board to test water monitoring and demonstrated improved results. On an application to assess agriculture water quality, Paepae (et al., 2021) [99] used a virtual sensing system to demonstrate physical sensor methods with clear results. These water quality sensors typically are exposed to environmental conditions such as rainfall, dust, and wind. To overcome this challenge, [63] developed a 3D printing system, with a method of fabrication which is durable in the long term. Brewin et al. 2019 [62], developed a pocket-size hand-held device with marine-resistant materials using a 3D printer to measure water clarity and colour in lakes, estuaries, and nearshore regions. Despite the fact that these innovations are brand new for IoT sensor applications for water-quality measurement, there is a lot of room for improvement in terms of artificial algorithms for accurate calibration.

## 4. Self-Calibration Techniques

Self-calibration techniques refer to methods and processes that enable a system, device, or instrument to automatically calibrate itself without the need for external reference standards or manual intervention [100]. They have been gaining attention in various fields, including engineering, metrology, and sensor technologies [100]. These techniques are particularly valuable in situations where traditional calibration methods may be impractical, time-consuming, or cost-prohibitive [101]. Self-calibration techniques are employed in various fields such as in-situ calibration, sensor fusion, machine learning calibration, and environmental monitoring [15,102,103,104].

Studies by [15,24,102,105,106,107] highlight the numerous benefits of self-calibration techniques. These advantages include real-time adjustment capabilities, allowing for continuous monitoring and adjustment of measurement equipment to maintain accuracy over time. Unlike traditional calibration methods, self-calibration reduces the need for human intervention, minimizing downtime and interruptions to equipment operation. Additionally, self-calibration enhances accuracy by continuously monitoring and correcting measurement deviations, reducing the risk of drift or changes between calibration sessions. Automation in calibration processes also decreases the likelihood of human error, ensuring more consistent and reliable calibration results. Moreover, self-calibration systems adapt to environmental changes and operating conditions, maintaining accuracy despite variations in external factors. While initial implementation may require investment, the potential for reduced manual labour and increased efficiency offers long-term cost savings.

Self-calibration methods vary depending on the type of sensor and the specific factors being addressed. For instance, the approach for self-calibration differs between chlorine sensors and sensors for gases like CO or HC [108]. These methods typically comprise several steps to ensure precise and dependable measurements. For example, zero calibration is the initial step, involving placing the sensor in a solution with zero chlorine concentration, such as deionized water, to establish a baseline reading. This calibrates the sensor to a reference point in the absence of chlorine. Subsequently, span calibration requires immersing the sensor in a known concentration of chlorine solution to calibrate its response at a specific chlorine concentration. Adjustments are then made by comparing sensor readings with expected values at zero and span calibration points, aligning the sensor output accordingly. Regular maintenance checks and calibrations are crucial for monitoring sensor accuracy over time, considering factors like sensor drift, environmental conditions, and aging. Some advanced chlorine sensors may provide automated calibration features or routines, necessitating adherence to the manufacturer’s instructions for effective utilization. Finally, verifying sensor performance involves testing it with known chlorine concentrations to ensure ongoing accuracy and reliability. These steps collectively ensure the precision and functionality of chlorine sensors for environmental monitoring applications.

Although there are multiple advantages in self-calibration methods, they do have some drawbacks in terms of accuracy, limited calibration range, and maintenance requirements [108]. It is important to recognize that the sensor’s properties undergo gradual changes over time, resulting in decreased accuracy. Therefore, regular calibration is essential to maintain precision. Additionally, determining the optimal calibration frequency depends on the specific application and is typically established through practical experience.

It is also crucial to acknowledge that self-calibration procedures may not be suitable for all applications or industries [109]. Traditional calibration methods, involving manual calibration by qualified specialists on a regular basis, remain prevalent and trusted in many industries [110].

## 5. Calibration Techniques and Developments in Their Accuracy

The design of an experiment and its sensor calibration methods may be more effectively directed if it is understood how LCSs differ from standard instruments. A widely reported issue with LCSs is that they suffer from large uncertainties relating to low data precision and accuracy [28,30,31,51]. This uncertainty in performance can be related to various limitations such as low-signal-to-noise ratios for different sensors, environmental factors, and low selectivity. Due to the different types of air quality sensors used, it is often difficult to compare data from different studies. For example, the same air quality parameter (PM2.5) was measured in similar sites in Nairobi, Kenya by Kiai et al. 2021, and Pope et al. 2018 [51,111]. However, there was a difference in the scaling factor used for calibration of the low-cost sensors and this can mainly be related to the differences in the type of low-cost sensors used [51].

In order to address these difficulties, LCSs may need extensive calibration processes. Sensor calibration is defined as ‘a process to determine the mathematical function (calibration function) that defines the relation between independent and dependent variable’. There are various methods used to obtain calibration data, for example, field calibration and laboratory calibration. For low-cost sensors, the calibration processes generally employed are automatic and semi-automatic techniques. The literature shows several methods that have been used to validate the calibrations obtained from LCSs, such as Tapered Element Oscillating Microbalance (TEOM), nephelometer, GRIMM EDM 180 monitor, and predominantly a gravimetric Federally Equivalent Method (FEM) instrument known as Attenuation Monitor (BAM).

Calibration models are applied during pre-deployment of sensors to deal with errors that occur in mapping raw sensor measurements; the fundamental methods here are offset and gain calibration [112]. ‘Gain’ describes the sensor’s response to rising pollutant concentrations, and ‘offset’ describes the sensor’s response to total absence of the target pollutant. In combination, they create the calibration curve.

For instance, Shi et al. 2021 [93] performed a simple one-point calibration before the sensor deployment (Equation (1)), the calibration offset here is the difference between air and depth sensors.

(1) $$d=\frac{p_{abs}-p_{air}-e_{cal}}{\rho_{water}\times g}$$

where *d* is water depth (*m*), *p_abs_* is the absolute pressure (*mbar*), *p_air_* is the ambient air pressure (*mbar*), *e_cal_* is the calibration offset (mbar) and *ρ_water_* is the density of water at a specific temperature (kg/m^3^), and g is the gravitational acceleration constant (9.81 m/s^2^).

There are other errors reported from the environment such as temperature, wind speed, relative humidity, and turbulence. Fang & Bate 2017 [28] studied cross-sensitive parameters between multiple parameters (Equation (2)) by adding the interaction terms into the calibration function. However, they concluded that using only a single parameter to calibrate low-cost sensors in urban environments is likely to be insufficient.

(2) $$Y=\beta_{0}^{\prime }+\beta_{1}^{\prime }\cdot X_{1}+(\beta_{2}^{\prime }+\beta_{3}^{\prime }\cdot X_{1})X_{2}$$

where *Y* is the dependent variable, *X*_1_ and *X*_2_ are independent variables which can be noise, and *β* is the calibration coefficient.

Data evaluation in LCSs typically includes outlier detection, inter-sensor comparisons, and comparison with traditional monitors [113], all of which contribute to data loss for a network, reducing the spatial resolution. Table 5 provides a summary of low-cost sensors from the literature review and the standard validation and accuracy methods employed in their studies. For instance, Fienberg developed a network of 20 sensor pods to study air quality at the Shelby Farm monitoring site, where three sensors never operated, six failed during operation, and with R^2^ threshold of 0.5, only six sensor pods met the data quality objective. Such sensor failure and data loss has been detected from other sensor networks as well [48,113,114,115]. Outlier detection is defined as the detection of values that are statistically significantly distinct from the other normal values at a given time and location [113]. Detection of outliers is a crucial element in finding erroneous values and removing them; they occur due to faults in sensors, weather patterns, and dirt attachment to sensors. For assessment of air-quality sensors, many calibration models, primarily Linear Regression (LR), Multivariate Linear Regression (MLR), or a variety of Machine Learning (ML) algorithms, including artificial neural networks, random forests, and support vector regression, among others, were used [29,116].

Water quality monitoring sensors are mostly deployed in water for a long period of time, therefore require protection from fouling/biofouling which leads to uncertainty in values [8]. Various studies [8,117,118] indicated that biofouling may be the cause for degraded water quality monitoring data, and their proposed solution is to design sensor nodes that are suitable for wiper cleaning. Another calibration uncertainty in water quality sensors is due to sensor drift, a temporal shift in the sensor’s response under constant physical and chemical conditions brought on by sensor damage from water pressure and water fluxes [119]. The literature showed that the major drawback concerning non-contact LCSs is practically observed [63] when the turbidity sensor stopped function after one month due to attachment of dirt such as mud/silt/microorganisms which need to be cleaned off regularly. Therefore, a sensor with a self-cleaning mechanism can improve the calibration results. Further, higher linearities for the signals received from sensors compared to actual measurements indicate a reduction in accuracy. Wong et al. 2021 [63] developed a 3D-printed water quality sensor which showed turbidity within the range of 10 to 1000 FNU and gives more accurate results, where optimum measurement ranges for the ultrasonic and temperature sensors are 2–400 cm and 10–50 °C, respectively. Calibration techniques mentioned in Table 5 provide insights into the calibration of both air quality and water quality sensors, including techniques such as MBE, RMSE, determination of correction factors of optical sensors using cyclone samplers, nephelometer, linear regression equation, MLR, LR, ANN, Mini-Vol configuration, correlations, ordinary least square regression, FRM, FEM, multiple linear regression, etc.

**Table 5:** Summary of low-cost sensors from the literature review and the standard validation and accuracy methods employed in their studies.

Author Ref.	Location	Calibration Methods Used	
		Standard Measurement Validation	Calibration Techniques
Air Quality			
**[44]**	Patras city in Greece	Compared with GRIMM EDM 180 monitor.	MBE, eMBE, rMAE, RMSE, R^2^.
**[51]**	Kenya	A standard Andersen dichotomous impactor (Sierra Instruments Inc., Monterey, CA, USA) was used to calibrate the low-cost sensors for four days by collocation.	Correction factor of the optical sensors (PMS7003) was determined from the cyclone samplers (BGI 400S).
**[52]**	Australia	The PurpleAir (PA-II) low-cost sensors were collocated with three reference sensors: Tapered Element Oscillating Microbalance (TEOM), nephelometer, DustTrak monitors.	TEOM sensors were related to the nephelometer using an equation. The low-cost sensors were calibrated (hourly and daily) using a relationship (equation) with the nephlometer and TEOM.
**[53]**	USA	TEOM federal equivalent method (FEM) monitor was used for reference sensor and collocation.	Linear Regression equation for the reference monitor (TEOM) and the low-cost sensors.
**[54]**	USA	A Met One Beta Attenuation Monitor (BAM); a gravimetric FEM instrument was used as the reference monitor and collocation.	Least squares linear regression, MBE, MAE.
**[55]**	USA, Rwanda, Malawi and DR Congo	BAMs were used as the reference monitor and collocation.	Linear regression, MAE.
**[29]**	Serbia	Automatic Monitoring Station (AMS) Stari Grad was used as the reference monitor and collocation.	NRMSE, LR, MLR, MBE and ANN Square Difference (uRMSD).
**[56]**	Palestine, Nablus	AirUs were calibrated for local PM using a filter-based, low-volume air sampler Mini-Vol configured for PM2.5 collection.	Mini-Vol configured for PM2.5 collection.
**[50]**	Australia	Correlation between the KOALA and TEOM over a 12-month period.	(R2 = 0.89), >0.90 for the daily averages between the TEOM and KOALA for PM2.5.
**[49]**	N/A	Fan-based.	Correlations (R).
**[120]**	USA	Calibration performed with reference to a reference ozone analyser (Thermo 49i), is manufactured by Thermo Fisher Scientific, located in Waltham, MA, USA	Ordinary least squares (OLS) regression.
**[121]**	China	Calibration and validation were performed with reference to US federal reference methods (FRMs; TEOM-FDMS, BAM, SHARP).	Linear regression, RH adjusted linear regression.
**[122]**	USA	Calibration and validation was performed with reference to federal equivalent method (BAM).	Multiple linear regression with the BAM PM2.5, RH, and T as predictors.
**[58]**	USA	Validation with reference to federal reference method (FRM).	Validation with reference to federal reference method (FRM) and federal equivalent method (FEM) monitors.
**[123]**	USA	Proxy model developed from a reference instrument.	Proxy corrected sensor data and K-means clustering.
**Water quality**			
**[61]**	Taihu Lake and Yuqiao Reservoir	Based on synchronous measurements with a field spectrometer, the results were validated.	RMSE, MRE, R^2^.
**[62]**	UK and destinations in the South Atlantic	An iButton temperature logger was attached to the mini-secchi disk and it was calibrated against a NIST-traceable (and NPL-traceable) Hart Scientific.	Comparison between the housed iButton and the NIST-traceable probe with the difference in average, median, absolute average, median absolute in N number of samples.

Linear regression (LR), Normalized Root Mean Squared Error (NRMSE), Multivariate linear regression (MLR), Mean Bias Error (MBE), Mean Absolute Error (MAE), and Artificial Neural Network (ANN), Coefficient of correlation (R), coefficient of determination (R^2^).

Most of the current studies utilizing low-cost sensors for air pollution measurement use simple linear regression to calibrate low-cost sensors in relation to the reference device to improve accuracy. However, linear regression cannot model this relationship since several non-linear and environmental variables can affect the accuracy of low-cost sensors [29]. Thus, to truly account for these variables, machine learning techniques may prove very useful [46,68,107].

There are several metrics that were employed to estimate the accuracy of sensor monitored values (Table 6). These metrics are designed to capture the main aspects of the time-series behaviours. The accuracy between these sensors and standard measures changes due to the seasons, location, and meteorological/water quality conditions of air/water. The accuracy of Plantower PMS7003 sensors was evaluated by Kiai et al. 2021 [51] and they showed that the sensor’s level of accuracy is high; earlier studies on Plantower sensors demonstrated better accuracies as well. These studies aid scientists and other interested parties in choosing a low-cost sensor for their research.

Rapid changes in meteorological conditions/water quality affects a sensor’s detected values and when the accuracy is assessed, the sensor shows large fluctuations [63,124].

From the literature, it can be seen that the calibration for any such LCS should be carried out using five primary indicators. They are (i) environment and its condition, (ii) pollutant/contaminant parameter(s) to be monitored, (iii) sensor specifications with its lower and higher accuracy range, (iv) validation instrument that was/were to be used, and (v) the type of regression model/models one uses to study the parameters [16].

## 6. Conclusions and Future Research Directions

This review article thoroughly explores the complex domain of low-cost sensors (LCSs), particularly those designed for monitoring air and water quality. The study also provides a brief insight into their advancements and barriers. Emphasizing the crucial contribution of LCSs to environmental research and public health, the discussion highlights their growing availability and cost-effectiveness as key facilitators.

Improved technologies and efforts in understanding environmental pollution are making low-cost sensors more available for research and understanding of the environment. The current study reveals that recent research has focused heavily on PM sensors in relation to air quality sensing. This may be due to the affordability of low-cost PM sensors and the growing public awareness of the health crisis caused by air pollution. However, these inexpensive sensors have faced significant difficulties due to the capital costs associated with installation, communication networks, maintenance, and the instruments for data interpretation [10].

The study also showed that, in contrast to air pollution, there is little research being done on the potential of water quality sensors. This may be as a result of a lack of regulations pertaining to water quality sensor technologies, as well as poorly defined categories of contaminants and exposure levels. The usage of LCSs in water quality applications, however, may rise as a result of growing concern over poor water quality and the cost benefits associated. To give good results, and to avoid malfunctioning, it is recommended to use sensors with a self-cleaning mechanism. For water quality measurement, the need for low-cost sensor devices with antifouling characteristics should be investigated and developed for commercial use [8], and additionally, standard algorithms for sensor drift should be developed [119]. However, these facilities demand a good power source and self-cleaning mechanism, which again is not adequately attainable with inexpensive sensors. For measurement of air pollution, several studies revealed that meteorological parameters such as relative humidity (RH), temperature (T), pressure (P), and wind impact the performance of low-cost sensors and therefore there is a need to use standard reference-grade monitoring stations for evaluation and validation. It is further advised to not rely on low-cost air-quality sensors at higher RH value locations [125].

In addition, the study also summarized self-calibration techniques and their contribution to enhancing accuracy, efficiency, and reliability. Calibrating low-cost environmental sensors presents both challenges and opportunities in the realm of air and water quality monitoring. While these sensors offer cost-effective solutions and increased spatial data density, they often suffer from uncertainties related to accuracy and precision. Challenges such as sensor drift, environmental factors, and limited calibration ranges underscore the need for robust calibration techniques. Various calibration methods, including automatic and semi-automatic techniques, have been employed to address these challenges, with a focus on offset and gain calibration models. Despite these challenges, advancements in self-calibration techniques and machine learning algorithms offer promising opportunities to improve sensor accuracy and reliability over time. Studies have highlighted the benefits of self-calibration, such as real-time adjustment capabilities and reduced reliance on manual intervention, leading to increased efficiency and cost savings in the long run. Additionally, machine learning algorithms have potential to act as a powerful tool for modelling complex relationships between sensor readings and environmental variables, enhancing the accuracy of low-cost sensor measurements.

The literature study further reveals that most of the correlations used were of the R square form and RMSE for measurement error analysis. Further, the accuracy of the sensors depends upon the environmental conditions, geological location, standard reference used, and the regression models [16]. Further, the literature review showed that Purple Air is the most used for air quality whereas many experiments on water quality have relied on IoT to monitor multiple parameters.

There is still a lack of regulatory bodies to maintain gathered data and oversee processing and usage of data for these sensors. Regular processing and maintenance of LCSs for commercial entities and scientific bodies is very challenging due to limited budgets. Citizen-owned networks with regulatory bodies may help to overcome part of this challenge.

To build confidence in low-cost sensors for real-world monitoring, regular calibration and validation with a co-located standard instrument is critical [125]. Improved statistical methods, IoT-based platforms, and space-based sensors can all improve methodological approaches for environmental pollution. Additionally, an inclusive approach using installed LCSs, standard measuring units, remote sensing, smart networks with effective communication, and data preservation can constitute best practice, while cutting-edge computational techniques like machine learning can make it easier to make a reliable forecast estimate for the future. For IoT sensors, an optimum maintenance time is required to ensure performance and cost-effectiveness of the system. Although there are still many issues related to LCSs that need to solved, their potential has been expanding due to the growing need for a clean environment and the climate change crisis in addition to the need to involve citizens in monitoring the local environment. The participation of citizens and their recognition of the obligation to maintain pollution metrics, such as indoor, outdoor air quality and water quality, has markedly expanded LCSs at the citizen level and is contributing to further advancements in sensor technologies.

However, it is essential to recognize that calibration procedures may not be suitable for all applications, and traditional calibration methods remain prevalent in many industries. Furthermore, ongoing research is needed to address the limitations of low-cost sensors, such as sensor drift and environmental factors, and to develop standardized calibration protocols for widespread adoption.

## Figures and Tables

**Table 1:** Inclusion and exclusion criteria used for the systematic review.

Criteria	Inclusion	Exclusion
Type of sensors	Low-cost sensors only	Not low-cost sensors
Type of study	Water and air quality studies	Other studies such as soil quality, UAV and photogrammetry
Calibration	Sensor calibration is done	Sensor calibration is not done
Field sensors	Sensors are used in the field	Sensors have not been used in the field
Language	English	Non-English

**Table 2:** Summary of articles considered for the review.

Sr. No	Ref.	Study Focus
Air Quality		
1	[37]	PM2.5 trend analysis using Beta Attenuation Monitor (BAM) and low-cost sensors Purple Air (PA) and Atmos low-cost PM2.5.
2	[38]	To study London’s air pollution and evaluate the uncertainties in 100 LCSs.
3	[39]	This study aims at testing Portable Air Pollution Sensors’ performance in the development of exposure surfaces (Traffic-Related Air Pollution) for nitrogen dioxide (NO_2_) and ozone (O_3_).
4	[40]	Used low-cost portable particle monitors for measurement of fine and coarse particulate matter in urban ambient air.
5	[41]	Developed an environmental chamber for evaluating the performance of low-cost air quality sensors under controlled conditions.
6	[42]	Investigated the PM2.5 and NO_2_ concentrations collected using low-cost sensors in Peñuelas, Puerto Rico.
7	[43]	Evaluated performance of inexpensive laser-based PM2.5 sensor monitors for typical indoor and outdoor hotspots of South Korea.
8	[44]	Studied the calibration process of low-cost sensors (PM) in ambient conditions.
9	[45]	Study was conducted to investigate particle size selectivity of low-cost sensors.
10	[46]	Evaluated the performance of a low-cost air sensor network for a period of approximately 18 month at the community scale.
11	[47]	Compared the results for PM from two devices: GENT stacked Filter Unit sampler and a microcontroller board with low-cost sensors.
12	[48]	Developed a hierarchical air quality measurement network, grounded in high-quality, compliant reference stations and extended to neighbourhood scale using low-cost sensors.
13	[49]	This study aims to define the applications of low-cost sensors (LCS) in measuring air pollutants and to show the effect of sensor place and car velocity in the performance of LCS.
14	[50]	Measured air quality after the commonwealth games in Australia and determined any impacts of the games.
15	[51]	Assessed the spatial extent and distribution of PM2.5 in Kenya.
16	[52]	Assessed the accuracy and potential of low-cost sensors in modelling the spatial distribution of PM2.5 and the population exposure to PM2.5 coming from domestic wood-heating.
17	[53]	Explored the potential of a network of low-cost air quality sensors through evaluation of spatiotemporal variability and sources of PM in the study area (Memphis).
18	[54]	Evaluated the field performance and accuracy of 12 low-cost sensors under the same ambient conditions.
19	[55]	Investigated satellite data (MODIS data) validity with low-cost sensors to improve the accuracy of both in measuring air quality.
20	[29]	Evaluated the accuracy of machine learning techniques for in-field low-cost sensor calibrations.
21	[56]	Measured PM2.5 and PM10 concentrations in three different locations in the city of Nablus by using calibrated low-cost sensors ((PMS) 3003, PMS 1003, PMS 5003, which are sourced from ‘Air sensor Toolbox’, United States Environmental Agency (Washington, DC, USA)
22	[57]	Determined PM2.5, O_3_, NO_2_ concentrations in Denver, Colorado using nine different air pollution sensors.
23	[58]	Measured air pollutants CH4, TNMHC, CO, and CO_2_ in Los Angeles, USA.
**Water quality**		
1	[59]	Leveraged simple, low-cost microprocessors, electronics, and housing components to design and construct open-source Optical Backscatter Sensors (OBSs).
2	[60]	Developed a cost-effective optical sensor for continuous in-situ monitoring of turbidity and suspended particulate matter concentration (SPM).
3	[61]	Used digital images to accurately calculate water leaving reflectance.
4	[62]	Evaluated a printable device which can measure the Secchi depth and water colour.
5	[63]	A 3D-printed IoT-based water quality monitoring system (WQMS) is developed and deployed, using only solar energy.
6	[64]	Developed a low-cost digital camera colorimetry setup to investigate quantitative relationships between water colour indices and concentrations of optically active constituents (OACs).

**Table 3:** Particulate matter characteristics.

Metrics	Equipment	Size Range	Detection Limits
Particle mass	Gravimetric filters	150 nm<	10 μg/m^3^<
	Photometer	(40–100 nm)–10 μm	0.001–200 mg/m^3^
	Low-cost photometers	N/A	0–600 μg/m^3^
Particle number	Condensation particle counter/CPC (full flow)	2.5–15 nm<	<1 × 10^4^–1 × 10^6^ particles/cm^3^
	CPC (mixing)	2.5–15 nm<	<1 × 10^4^–1 × 10^6^ particles/cm^3^
	Optical particle counter/OPC	0.3–20 μm	<1 × 10^4^ particles/cm^3^
	Diffusion size classifier/DiSC	10–700 nm	<5 × 10^2^–1 × 10^6^ particles/cm^3^
Particle size distribution	Impactors	1 μm–10 μm	N/A
	Scanning mobility particle sizer/SMPS	2.5–1000 nm	1–1 × 10^7^ particles/cm^3^
	Aerodynamic particle sizer/APS	0.5–20 μm	1000 particles/cm^3^
	Fast mobility particle sizer/FMPS	5–560 nm	N/A
Particle surface area	Nanoparticle surface area monitor/NSAM	10–1000 nm	<10,000 μm^2^/cm^3^

**Table 4:** Various low-cost sensor models used for air-quality monitoring from the literature.

Author Ref.	Sensor/Model	Cost	Detected/Detectable Parameters
**[37]**	PurpleAir (PA-II-SD) and Atmos	PA-II-SD: USD 299, Atmos: USD 369	PA-II-SD: PM2.5, pressure, temperature and humidity; Atmos: PM1, PM2.5 & PM10 µg/m^3^, Temperature, Relative Humidity
**[38]**	AQMESH v. 3.5 units	USD 173.40	CO, NO, NO_2_, O_3_, Temp, RH
**[39]**	Aeroqual portable monitors, S500	-	NO_2_, O_3_
**[40]**	Dylos DC 1700 PM sensor	USD 400	PM2.5 and PM10
**[42]**	OEM sensors/OPC-N2 Particle Monitor/Mocon piD-TECH/CairPol CairClip	<USD 2500	The CairClip useS a gas-specific inlet filter combined with dynamic air sampling in an integrated system to measure real-time NO_2_ concentrations with a detection limit of 1 ppb; the OPC-N2 uses light particle counting to measure the concentration of suspended particles in the air sampled via an internal pump and has a detection limit of 0.1 µg/m^3^
**[43]**	GRIMM180 (GRIMM Aerosol, Ainring, Germany)/ESCORTAIR (ESCORT, Seoul, Republic of Korea)/PurpleAir (PA)	<USD 300	The performance of the two IRMs (one OPC, that is, ESCORTAIR and one photometer, that is, PA) costing less than USD 300 were simultaneously compared with those of high-cost devices (USD 10,000 or so), that is, research-grade laser photometers including PDR-1500 (Thermo Scientific, Waltham, MA, USA) and SIDEPAK AM510 (TSI, Inc., Shoreview, MN, USA)
**[44]**	PMS 5003 sensors and GRIMM EDM 180 dust monitor	USD 22.76	PM1, PM2.5, PM10 mass concentrations
**[45]**	Plantower PMS5503, Nova SDS011, Sensirion SPS30, Sharp GP2Y 1010AU0F, Shinyei PPD42, Omron B5W	-	PM1, PM2.5, PM4, PM10
**[46]**	PurpleAir II	-	PM2.5
**[47]**	A microcontroller board and low-cost sensors including dust sensor, smoke sensor, liquefied petroleum gas sensor, carbon dioxide (CO_2_) sensor, carbon monoxide (CO) sensor, temperature, and humidity sensors	-	PM2.5
**[51]**	Plantower PMS7003 sensors and cyclone samplers (BGI 400S)	USD 30 for the PMS7003 sensors	PM2.5
**[52]**	PurpleAir (PA-II) units	<USD 300	PM2.5
**[53]**	Alphasense OPC-N2 sensor	-	PM2.5, wind direction, temperature and relative humidity (RH)
**[54]**	Shinyei PM Evaluation Kit, Alphasense OPC-N2, TSI AirAssure, Hanvon N1, Airboxlab Foobot, Kaiterra LaserEgg, PurpleAir PA-II, HabitatMap Air Beam 1, SainSmart Pure Morning P3, IQAir AirVisual Pro, Uhoo and Aeroqual AQY	USD 1000, USD 450, USD 1000, USD 200, USD 200, USD 200, USD 230, USD 200, USD 170, USD 270, USD 300, USD 3000	PM2.5
**[55]**	MetOne NPM, PurpleAir PA-II, and Alphasense OPC	USD 2000, USD 250, USD 350	PM2.5
**[29]**	AQMESH v. 3.5 units	NA	NO_2_, NO, CO, O_3_, PM2.5, PM10, temperature, relative humidity and pressure
**[56]**	Plantower Particulate Matter Sensor (PMS) 3003, PMS 1003, PMS 5003	USD 22.93, USD 19.24, USD 21.45	PM2.5 and PM10
**[57]**	Aeroqual SM-50, TSI AirAssure, AirCasting AirBeam, Cairpol CairClip, Dylos DC1100/DC1100 Pro, AlphaSense OPC-N2, Shinyei PMS-SYS-1, AirViz Speck, TZOA PM Research sensor.	N/A	PM2.5, O_3_, NO_2_
**[58]**	Single Sensor—CH_4_, Multi-sensor—CH_4_, Multi-sensor—TNMHC.	N/A	CH_4_, TNMHC, CO, CO_2_
**[50]**	9 low-cost sensors (KOALA)	N/A	PM2.5, CO
**[76]**	Teledyne T400, Teledyne T640	N/A	PM2.5, O_3_
**[55]**	Met-One NPM (25 sensors), PurpleAir PA-II (9 sensors)	PurpleAir sensors: (sub-USD 250 each), NPM: (sub-USD 2000 each)	PM2.5
**[77]**	Random forest (RF) model	N/A	O_3_, NO_2_

EUR converted into USD at EUR 1 = USD 1.08.

**Table 6:** Summary of accuracy assessment formulae from literature.

Ref.	Description	Formula
Air Quality		
**[51]**	Accuracy assessment.	$A=100-\frac{\left|X-R\right|}{R}\times 100$ where A = accuracy %, X = average concentration for sensors, and R = average concentration for reference sensor. Using this equation, they calculated the accuracy of their low-cost sensors to range from 81.47% to 98.60%.
**[52]**	Calibration of low-cost sensor using reference sensor.	neph = 0.023 × PA_2.5_ + 0.03 (hourly averages) where neph = nephelometer measurements, PA_2.5_ = PurpleAir measurements. The correlation; R^2^ = 0.99.
**[54]**	The measurement and accuracy of the twelve sensors were evaluated against a reference instrument for three years using Least squares linear regression, Mean Bias Error (MBE) and Mean Absolute Error (MAE).	The linear equation used is Y = mX + b where Y = PM_2.5_ measurement for a low-cost sensor (1-h average), X = PM_2.5_ measurement for the reference sensor; Met One Beta Attenuation Monitor (1-h average). m = slope and b = intercept. Only six of the twelve sensors had an average correlation; R^2^ ≥ 0.70.
**[57]**	Linear regression between the reference monitor (TEOM FEM monitor) and seventeen low-cost OPC-N2 sensors.	Y = mX + C where Y = measurements from low-cost sensors and X is the measurement from the reference sensor. Only six of the seventeen deployed OPC-N2 sensors achieved regression R^2^ > 0.5.
**Water quality**		
**[63]**	Linear regression between Standard formazin solutions (reference) and turbidity sensors using the ratiometric method.	Y = 0.001x + 0.0116 where Y = low-cost sensor signals and x = reference signal. The correlation; R^2^ = 0.992.
**[63]**	The actual distance of 2–400 cm of water level was compared with the distance measured by the sensor.	Y = 1.0119x + 0.5114 where Y = actual distance (from 2–400 cm) and x = distance measured by sensor (from 2–400 cm). The correlation; R^2^ = 0.9999.
**[63]**	Low-cost sensor temperature values were compared with analogue signals transmitted by the temperature sensor.	Y = 1.9689x + 122.92 where Y = Analog signal and x = Observed temperature. The correlation; R^2^ = 0.9936.

## Data Availability

Data are contained within the article.
